# Skimboarding: An Increasingly Recognized Cause of Life-Threatening Spinal Cord Injury

**DOI:** 10.7759/cureus.20915

**Published:** 2022-01-03

**Authors:** Ana Marta Mota, Cícero J Silveira, José J Nóbrega, Pedro S Lima

**Affiliations:** 1 Intensive Care Department, Hospital Central do Funchal, Serviço de Saúde da Região Autónoma da Madeira, Entidade Pública Empresarial da Região Autónoma da Madeira, Funchal, PRT; 2 Neurosurgery Department, Hospital Central do Funchal, Serviço de Saúde da Região Autónoma da Madeira, Entidade Pública Empresarial da Região Autónoma da Madeira, Funchal, PRT

**Keywords:** water sport injuries, skimboarding, traumatic cervical spine injury, life-threatening, spinal cord

## Abstract

Skimboarding is a sport that combines elements from aquatic and terrestrial sports and has gained popularity with increased riskier maneuvers. Spinal cord injuries associated with this sport have rarely been reported. Here we present a case of a previously healthy 44-year-old male with a life-threatening C2/C3 fracture and dislocation after a skimboarding fall. Traumatic facet dislocations in the cervical spine are usually consequent to high-energy transmission injuries, so it is difficult to explain the mechanism of injury in this clinical case. As this sport’s practice continues to grow, our purpose is to emphasize that these injuries may occur with irreversible consequences as most of the damage occurs at the time of presentation, so the first step is to alert athletes and the community to prevent them.

## Introduction

The incidence of spinal fractures related to trauma is about 19-88 per 100,000 persons, 19%-51% of which involve the cervical spine, and cervical spinal cord injuries are associated with a much higher short- and long-term morbidity than other spinal cord injuries [1,2]. In comatose trauma patients, the prevalence of cervical spinal cord injury accounts for 7.7%, almost half of which are unstable injuries [3]. Incomplete spinal cord lesions are the most frequent injuries (31%), and it is estimated that tetraplegia occurs in 20% of the cases [2]. Motor vehicle collisions are the leading cause of spinal cord injury (42%), and sports injuries are a less frequent cause (8%) [2,4]. Injuries of C2-C3 are rare, as a result of the biomechanics of the vertebral column [5]. Many of these patients die at the scene due to their injuries and 29% die before reaching the hospital due to other injuries or respiratory muscle paralysis [6].

Skimboarding is a sport that combines elements from aquatic and land-based sports [7]. It is performed in shallow water, where the wave recedes towards the sea. This sport has gained popularity and has also increased difficulty with extreme and riskier maneuvers. Extremity fractures, mostly of lower limbs, are usually associated with skimboarding, but spinal cord injuries have rarely been reported [8]. This case has a straightforward clinical presentation - acute neurological dysfunction below the injury level - but it is a very rare mechanism of injury consequent to this type of water sport.

## Case presentation

**Figure 1:**
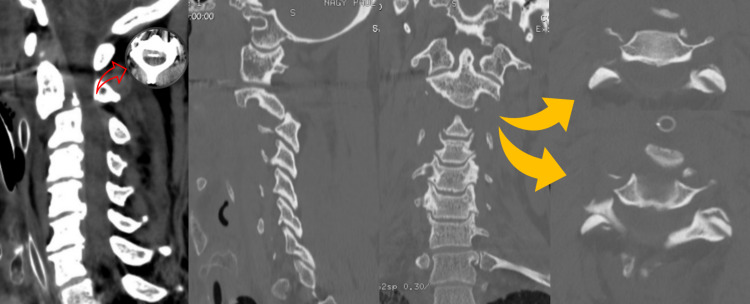
Cervical CT scan Left to right: Sagittal soft tissues (red arrow shows spinal cord compression between the C2-C3 segment), sagittal bone, coronal, and axial (yellow arrows show dislocated facets, reverse “hamburger bun” sign)

**Figure 2:**
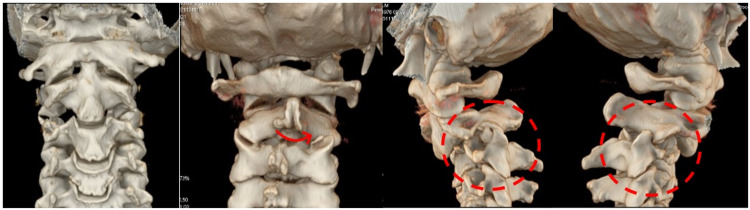
3D cervical CT scan Left to right: Translation (arrow) and bilateral fracture and dislocated facets (red circles).

A previously healthy 44-year-old male was admitted to the emergency department after a cardiorespiratory arrest while skimboarding. The fall mechanism was unknown, but a relative and other people nearby noticed the patient asking for help at sea, very close to the sand. He was taken out of the water as he couldn’t move his legs, was having difficult on breathing although still had a pulse but quickly evolved to cardiorespiratory arrest. Basic life support was promptly started at the scene and continued on for approximately 20 minutes. When the pre-hospital team arrived on the scene, the patient had already recovered spontaneous circulation, and they proceeded to spine immobilization and orotracheal intubation. During the transport, he became hypotensive and was started on vasopressor support. He arrived at the emergency department hemodynamically stabilized although neurological status was not assessed due to the need for ventilation under sedation. There weren’t other traumatic lesions. Blood tests, electrocardiogram, and brain CT scan were normal. Cervical CT scan (Figure 1) showed an unstable C2/C3 fracture and dislocation with spinal cord and C3 root compression - AOSpine type C, subtype F4 Nx/M2 (Figure 2). Vertebral arteries were not affected.

**Figure 3:**
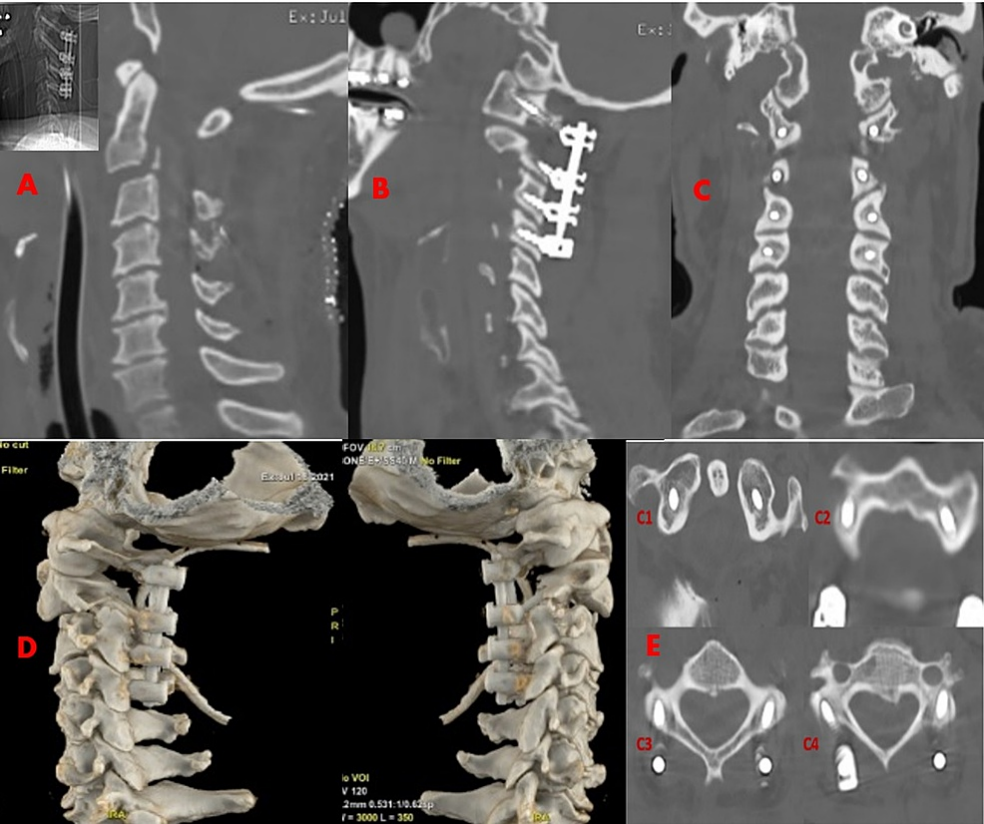
Postoperative cervical CT scan (bone) Sagittal and coronal (A-C), axial (C1-C4), and 3D (D-E).

Patient’s initial neurological evaluation, still while being sedated, showed a positive Babinski signal (bilateral), so he was promptly submitted to posterior cervical approach: decompression (C2 laminectomy, C2-3 facetectomy) and stabilization by C1-C4 fixation with screws and bars (Figure 3).

**Figure 4:**
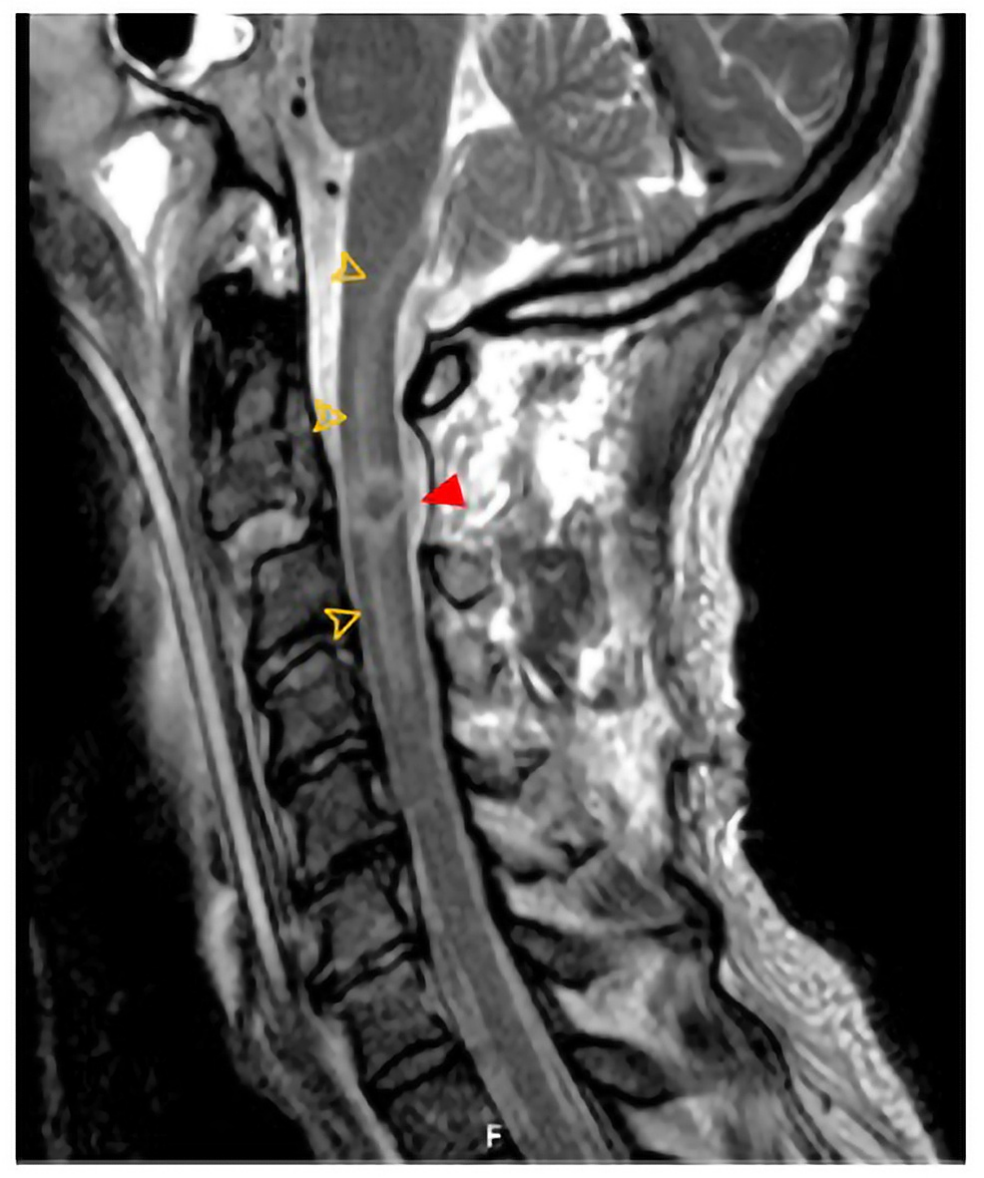
Postoperative cervical MRI scan (sagittal) The scan shows effective spinal cord decompression but important spinal cord contusion at C2-C3 (red arrowhead) due to important adjacent swelling until medulla (yellow arrows). MRI, magnetic resonance imaging

After surgery, he was admitted to the intensive care unit and the CT scan performed 24 hours after admission demonstrated effective spinal cord decompression in C2 with appropriate posterior cervical stabilization (lateral mass C1, C3, and C4; transpedicles C2). About 72 hours later, a cervical MRI scan was performed and showed spinal cord edema and contusion (Figure 4). The patient was now alert, attentive and oriented but remained in neurogenic and spinal cord shock for several days. Patients with cervical spinal cord injuries are at high risk of developing neurogenic shock as they suffer an interruption of the sympathetic chain resulting in unopposed vagal tone. In this case, despite the vasopressor support and fluid resuscitation, the patient had several short episodes of extreme bradycardia and cardiorespiratory arrest. Hence, he needed a temporary pacemaker for a few days. This lesion also resulted in a loss of diaphragmatic innervation and a loss of chest and abdominal wall strength. Therefore, the patient was tracheostomized because of ventilator dependency with difficulty on weaning. After approximately one month, he recovered sensitivity in the sacral segments, but tetraplegia was observed (American Spinal Injury Association [ASIA] scale B).

Almost three months after cervical surgery, he repeated a cervical CT scan that did not show complications or need for new surgery with partial consolidation of screws, but the neurological status had kept, i.e. tetraplegia below the C4 segment.

## Discussion

The annual incidence of subaxial cervical spine fractures is 10 per 100,000 persons. The prevalence of spinal trauma associated with water sports has increased in the last 10 years and it is more often associated with spinal cord injuries than vertebral trauma in a terrestrial environment [9].

Skimboarding can be practiced competitively or in a ludic way. To board, the rider must run, throwing the board, jumping on and riding parallel to the beach. He can also ride away from the shore until encountering an incoming wave, where he then changes directions and rides the tide back to the shore [7]. The surface of the skimboards is much smaller than the surfboards, which means that skimboarders have to use strategies to stay more time afloat, like pump when they reach breakers, i.e. stomp their front foot up and down on the top of the board. Skimboarding is perfectly practiced in shallow water because there is less water to move [10].

Many studies have demonstrated the association between skimboarding and lower extremity fractures, but spinal cord injuries are rarely described [11-15]. Collier et al. reported three cases of spinal cord injuries resulting from skimboarding accidents: two with C5 fracture with some motor skills restored, and one with C3/C4 fracture that remained ventilator dependent [8]. Kane et al. evaluated the epidemiology of water sports injuries at a coastal tertiary trauma center to determine its association with spinal column injuries and compare with those occurring terrestrially. They found a high incidence of vertebral column and spinal cord injuries in water sports, with a significant risk of cervical injuries [16]. Therefore, traumatic cervical injuries while practicing this type of water sport are rare. In this clinical case, it is difficult to explain the mechanism of an injury that impacts the passage of different fluids (air/water) and at a low height from a solid surface, in a young person. Also, traumatic vertebral injuries in low-energy trauma (e.g., falls from a standing height) are more common in the elderly due to degenerative and bone structure changes than in young individuals.

**Figure 5:**
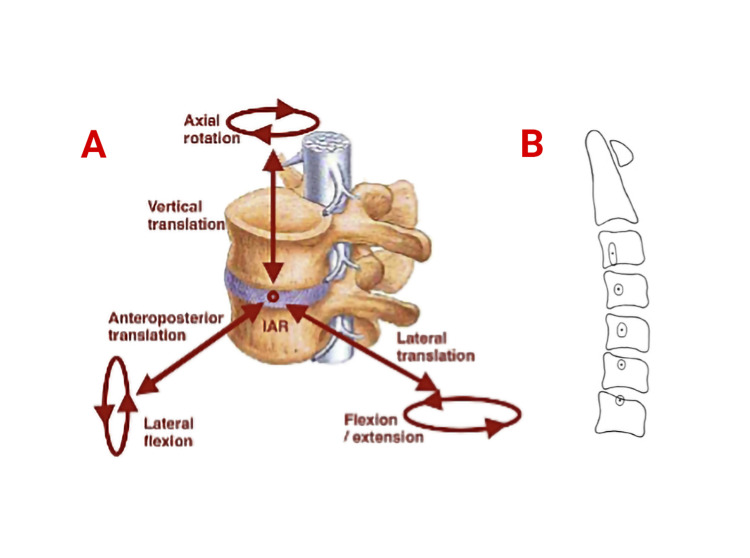
Spinal movement (A) Cartesian coordinate system with the IAR as the center. Translation and rotation can occur in both of their respective directions about each axis. (B) A sketch of an idealized cervical vertebral column illustrating the mean location and a two standard deviation range of distribution of the IAR of the typical cervical motion segments. IAR, instantaneous axis rotation Adapted from Loughenbury et al., with permission from the original publisher [9].

The most common injury mechanism of traumatic facet dislocations is flexion-distraction. The classification of the injury mechanism combines morphological changes in the spine with a force vector resulting in a point of application in the spine (e.g., AOSpine) that does not consider the kinematics (momentum). A vector force here may act on a lever, causing a bending moment that when applied to the point of the spine, causes rotation about an axis. This axis is named the instantaneous axis of rotation (Figure 5) [17]. Bones, ligaments, and tendons are all viscoelastic materials, so the speed at which the force is applied alters their mechanical behavior and ultimate tensile strength [9].

In the authors’ opinion, the most exciting aspect in this clinical case was to try to understand what kind of mechanism of lesion provoked this accident. Maybe combined forces, i.e. momentum and bending moment and the skimboarding being practiced in shallow water, essentially on the land, took sides in this event. The truth is that competitivity has scaled up in this sport making riskier maneuvers more common, which increases the risk of significant injury from landing headfirst in shallow water [8].

Early decompression (<8 h compared to >72 h) has been associated with decreased levels of tumor necrosis factor (TNF-α) and fewer apoptotic cells in injured spinal cord tissue, and these factors were associated with improved neurologic recovery [18]. This young patient had no morbidities, an indeterminate neurological status, a cardiorespiratory arrest at the accident site, and was admitted to the emergency department within three hours of the trauma. Also because of these reasons, he underwent early surgery. To manage cervical facet injuries, both anterior and posterior approaches are effective. The primary contraindication to the posterior approach to cervical trauma is ventral pathology such as intervertebral disc fragment or bony retropulsion compressing the anterior spinal cord. In subaxial facet dislocations, the initial management is closed reduction as soon as medically appropriate. After reduction, surgical stabilization is usually necessary because up to 40% of cases remain unstable after three months of halo immobilization. The best choice depends on the morphology of the injury, patient factors, and surgeon preference. The posterior approach allows for easier open reduction and biomechanically superior fixation. In this case, the patient didn’t need a second cervical surgery.

It is difficult to estimate the future prevalence of spinal cord injuries related to skimboarding, although as the sport continues to grow and evolve, an increase in these kinds of lesions can be expected [8]. Besides the improvements in medical care and rehabilitation, the average life expectancy of the patients with traumatic cervical spine injuries continues to remain lower than for the general population, and this is profoundly affected by the degree and level of injury. It is essential to highlight that most of the damage occurs at the time of presentation and is immediately irreversible, so the first step is to be alert and try to prevent this kind of injury [4,8].

## Conclusions

The knowledge about life-threatening spinal cord injuries due to skimboarding is low, and the risk of these lesions may increase in the following years as skimboarders continue to try more extreme maneuvers. This clinical case shows that it is crucial to conjugate the three pillars: pre-hospital stabilization, early surgical approach, and intensive care management. It is necessary to understand the mechanisms for lesions in this subtype of aquatic sport and health professionals must become more aware of the risk of injuries to improve the diagnosis and management of these patients. Furthermore, the skimboarders, their families, and general society must bear in mind the risk associated.
